# Current status and influencing factors of digital health literacy among community-dwelling older adults in Southwest China: a cross-sectional study

**DOI:** 10.1186/s12889-022-13378-4

**Published:** 2022-05-17

**Authors:** Siqi Liu, Hongyan Zhao, Jingjing Fu, Dehui Kong, Zhu Zhong, Yan Hong, Jing Tan, Yu Luo

**Affiliations:** 1 School of Nursing, Army Medical University (Third Military Medical University), No. 30 Gaotanyan Street, Shapingba District, Chongqing, 400038 P. R. China; 2 Xiaolongkan Community Health Care Service Centre, No. 4 Xiaolongkan Street, Shapingba District, Chongqing, 400030 P. R. China

**Keywords:** Digital health literacy, eHealth literacy, Internet use, Internet health information, Older adults, China

## Abstract

**Background:**

The rapid development of digital health has reduced the time and cost of medical treatment, bringing efficient and economical benefits. However, older adults all over the world are deficient in digital health knowledge and skills to varying degrees. This study intends to investigate the current status and influencing factors of digital health literacy among community-dwelling older adults in Southwest China, so as to provide theoretical reference for global digital health researches and the construction of gerontological digital health service models.

**Methods:**

A cross-sectional survey was conducted from September 2020 to April 2021 in Chongqing, China. 572 community-dwelling older adults (≥ 65 years) were surveyed by stratified sampling. Data on sociodemographic characteristics, Internet usage, attitude towards Internet health information and digital health literacy were collected. Wherein, the digital health literacy assessment adopted the Digital Health Literacy Assessment Scale for community-dwelling older adults, which was developed by the research group, proven to be with good internal consistency (0.941), split-half reliability (0.889), test–retest reliability (0.941), content validity (0.967), criterion validity (0.938) and construct validity. The influencing factors were explored by univariate analysis and multiple linear regression analysis.

**Results:**

The average score of digital health literacy was 37.10 (SD 18.65). Univariate analysis showed that there were statistically significant differences in the comparison of digital health literacy according to 16 variables, such as different age and education levels. Multiple linear regression analysis showed that education level, marital status, self-rated health status, degree of health concerns, duration of Internet usage, time spent using the Internet per day, frequency of Internet usage, frequency of receiving guidance passively from family members, perceived usefulness, perceived ease of use and perceived reliability were positively correlated with digital health literacy, while age and perceived risk were negatively correlated with digital health literacy.

**Conclusion:**

The overall digital health literacy of community-dwelling older adults in Southwest China is relatively low. In the future, health professionals should fully consider the diverse influencing factors of digital health literacy, assess individual differences and provide targeted intervention programs. Meanwhile, global public health authorities should integrate health resources effectively, and seek health service models for older adults in line with the development of the digital age to narrow the digital divide.

**Supplementary Information:**

The online version contains supplementary material available at 10.1186/s12889-022-13378-4.

## Background

According to the report of Internet World Statistics, by the first quarter of 2021, the number of global Internet users is over 5.1 billion, with Internet penetration rate of 65.6% [1]. With the development of Internet, information and communications technology (ICT) drives the constant innovation and upgrading of medical service models, and more and more health resources are available online, providing the public with a new method of seeking health information, facilitating health communication and treating diseases [2–4], namely e-health [5, 6], also known as digital health [7]. During the COVID-19 pandemic, the importance of digital health has become more prominent [8]. To reduce the risk of cross-infection in offline medical treatment, many governments encouraged online medical treatment, home delivery of medicine and online medical insurance services [9], which promoted the further development of digital health.

Digital health has become an important way to achieve the vision of health for all and has proven to be beneficial for both individuals and society [10]. From an individual perspective, digital health services help to increase public participation, promote better self-care behaviors and healthy lifestyles [11, 12], and thus improving health outcomes [13, 14]. From a socioeconomic perspective, the use of digital health can generate sustainable economic net benefits by providing better treatment for patients and improving the efficiency of patient management [15].

While fully enjoying the convenience brought by the digital dividend, we should pay more attention to the digital divide and health inequality faced by older adults as vulnerable groups in the digital world. The aging process of the world population is accelerating. It is estimated that by 2050, the world's population aged 60 years and older will total 2 billion, up from 900 million in 2015 [16]. Older adults generally suffer from a variety of chronic diseases, and health self-management is one of the important means to improve their health status. Especially community-dwelling older adults, compared with institutionalized older adults, they have stronger independence, need more health information, to optimize opportunities for health, participation and security with the aim of enhancing the quality of life, which is the reflection of active aging [17]. The emerging digital technology offers new opportunities to achieve the goal. However, previous studies have shown that the acceptance and application of digital health by older adults is very limited [18, 19], which is reflected in the difficulty in navigating the computer/Internet, knowing which resources to trust, logging into patient portals, physical limitations that reduce accessibility (e.g., difficulty reading text on screens), and privacy/security concerns, etc. [13].

The ability to use digital health, that is, digital health literacy, is defined as "the ability to search, understand and evaluate health information on digital media, actively participate in health information exchange and interaction, and meanwhile use the obtained information to address or solve health problems" [20, 21]. The lack of digital health literacy has become the main obstacle that hinders older adults from integrating into the digital society and enjoying convenient and efficient digital health services. Besides, older adults with a low level of digital health literacy when exposed to myths and rumours about COVID-19 abound, likely to become a marginalized but more vulnerable group [22]. Uncertainties regarding the quality of Internet health information not only created negative emotions like anxiety and panic [23], but also had a harmful influence on their health-related decisions [24]. Due to the above reasons, digital health literacy of older adults has gradually attracted the attention of scholars and then series of studies have been carried out in recent years. EHealth literacy Scale(eHEALS) is the most widely used digital health literacy assessment tool at present, which was developed by Norman and Skinner in 2006 [25]. Most of the existing studies used this scale to evaluate the digital health literacy of older adults [13, 26, 27]. However, it still has some problems, such as unclear score boundary [28], inability to accurately judge the actual level of users' digital health literacy [29], and lack of evaluation of critical and interactive health literacy [30, 31]. Stellefson proposed that with the rapid development of digital health, older Internet users’ online health information behavior is also changing, so it is necessary to modify eHEALS when it is applied to older adults [32]. At the same time, the existing researches on influencing factors of digital health literacy among older adults mainly focus on objective factors such as sociodemographic characteristics and digital factors [13, 33, 34]. Less attention is paid to subjective factors such as attitude towards Internet health information and social environment factors such as family support, which is lack of systematicness and integrity.

China is the largest developing country and the largest digital society in the world, with a population of 989 million Internet users (penetration rate 70.4%), which account for about 1/5 of the global Internet users [1]. China has witnessed the rapid popularization of mobile phones and the fast development of digital health technology. In the last decade, the Chinese government has been committed to actively promoting the adoption of ICT into the healthcare domain [35, 36], and meanwhile it has also introduced a series of policies to improve the digital adaptation of older adults [37]. However, at present, the lack in the knowledge of the overall digital health literacy level of community-dwelling older adults in China virtually hinders the long-term development of digital health and smart elderly care. In the early stage, based on the background of the current digital age of health care, our research group has developed a localized digital health literacy assessment scale for community-dwelling older adults by combining the current context of the Internet in China and the characteristics of digital devices usage among older adults. The scale has been proved to have good discrimination, reliability, validity and practicability, which provides an objective evaluation tool for evaluating the digital health literacy of older adults accurately and comprehensively. On this basis, the expected objectives of this study are proposed as follows: 1) Use the Digital Health Literacy Assessment Scale to assess the status quo of digital health literacy of community-dwelling older adults, and clearly depict the digital health usage scenarios of community-dwelling older adults in China. 2) Analyse the influencing factors of digital health literacy of community-dwelling older adults by comprehensively considering the sociodemographic characteristics, previous Internet usage and attitude towards Internet health information.

## Methods

### Participants and procedure

**Fig.1:**
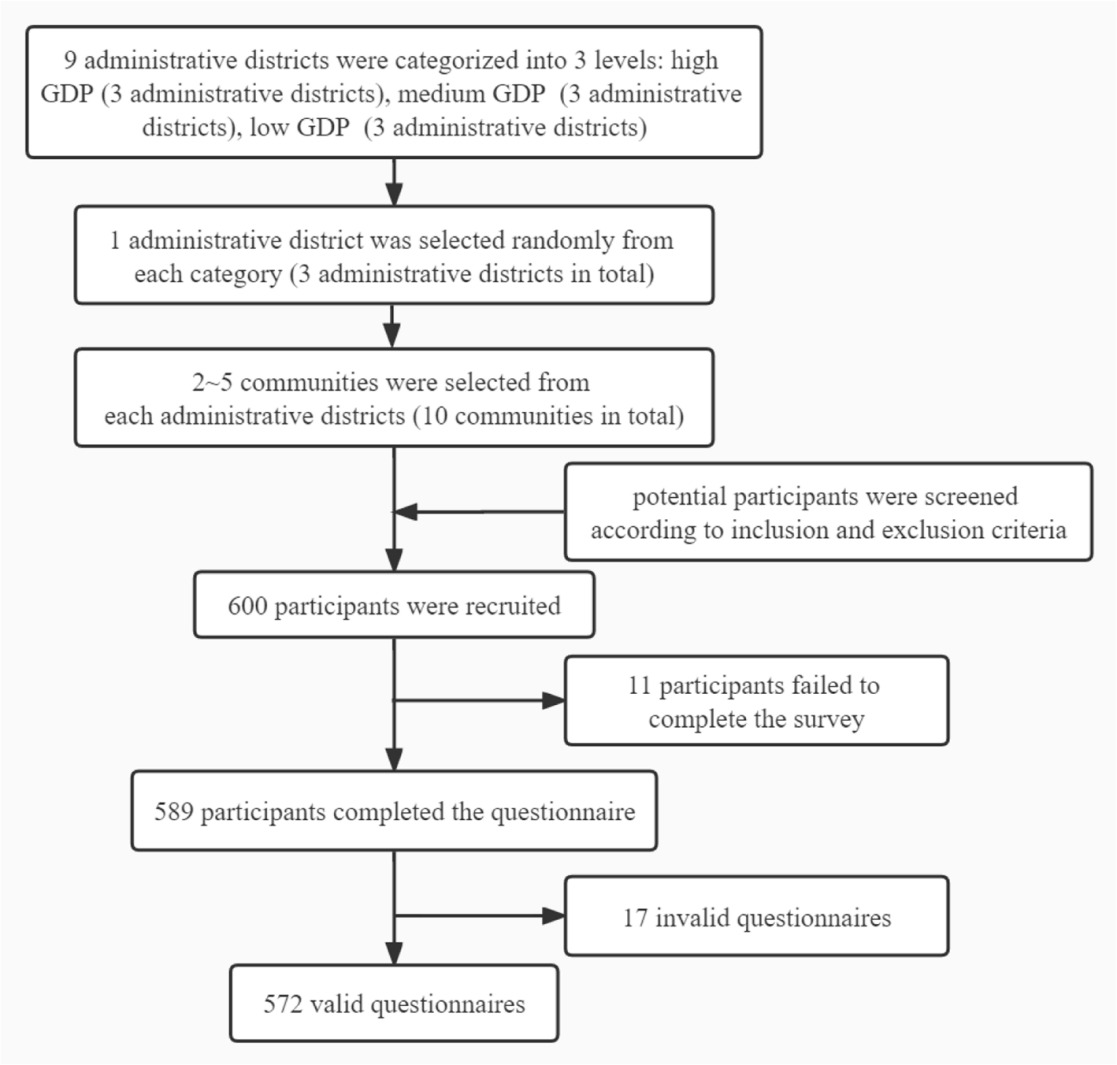
Participant enrollment procedure

This study was a cross-sectional study, which was carried out in the main urban areas of Chongqing, China from September 2020 to April 2021. As a megacity in Southwest China, Chongqing's population of those over 65 was 5.4749 million, accounting for 17.08% of the total population, which was higher than the national average of 13.50% of that population [38]. The sample size was calculated using the following formula: *n* = [Z^2^_α/2_p(1-p)]/δ^2^, which is commonly used in cross-sectional studies [39]. In previous studies, the qualified rate of digital health literacy among older adults in China was 11.1% [40]. Thus, δ was set to be 0.11 in this study. Using α = 0.05, Z_α/2_ = 1.96, δ = 0.03, our estimated minimum sample size was 418. The subjects were selected by stratified sampling. Figure 1 shows the sampling procedure. In the first stage, Chongqing’s 9 administrative districts in main urban areas were categorized into three levels according to their gross domestic product (GDP) per capita, and 1 administrative district was randomly selected from each category. In the second stage, 2 ~ 5 communities were selected from each of the selected administrative districts. In the third stage, old adults who met the inclusion criteria were recruited from the selected communities for a questionnaire survey. The inclusion criteria of the study population were: aged 65 years and above; having lived in the main urban areas of Chongqing for more than 6 months; voluntary participation. Exclusion criteria of the study population were: those with mental illness or confusion; those with a major illness that makes them unable to cooperate.

Research group members who had received standard training served as investigators, explaining the purpose and significance of the research to the participants, and distributing paper questionnaires after obtaining written and oral informed consent. Questionnaires were filled out by older adults themselves. For older adults who had difficulty in filling in the forms, the investigators read the questionnaire to them item-by-item and then recorded their responses. In the end, A total of 600 questionnaires were distributed and 589 questionnaires were collected. After excluding 17 invalid questionnaires (such as continuous repetition, regular answers or logical contradictions, etc.), 572 valid questionnaires were obtained, with an effective recovery rate of 95.33%. The study was conducted in accordance with the Declaration of Helsinki. Our research was ethically approved by the Ethics Committee of Army Medical University/Third Military Medical University (approval number 2020–012-02).

### Measures

The questionnaire consisted of three parts (see Additional file 1 for full questionnaire), which was developed based on a review of the literature on Internet health information seeking and digital health literacy. In order to ensure that the questionnaire is scientific and reasonable, we invited 6 experts to evaluate the content validity of the questionnaire. The content validity of each question was rated on a 4-point Likert scale (1 = not relevant to 4 = highly relevant). The item-level content validity index (CVI) was computed as the proportion of experts who rated a question as quite or highly relevant, and the scale-level CVI was computed as the average of all item-level CVI [41].

#### Sociodemographic characteristics

The tool used to measure the sociodemographic characteristics comprises 9 items, including gender, age, education level, monthly household income per capita, residential status, marital status, self-rated health status, degree of health concerns and chronic diseases.

#### Internet usage and attitude towards Internet health information

The tool used to measure the Internet usage characteristics and attitude towards Internet health information comprises 9 items. Participants were asked to provide details about their previous Internet use experience, including duration of Internet usage, frequency of Internet usage, time spent using the Internet per day, frequency of asking for help actively from family members on acquiring Internet health information, and frequency of receiving guidance passively from family members on acquiring Internet health information. Attitude towards Internet health information were assessed by rating the perceived usefulness/ease of use/risky/reliability of Internet health information. The item-level CVIs of all questions were rated 1.000, and the scale-level CVI thus computed was also 1.000.

#### Digital health literacy assessment scale for community-dwelling older adults

The scale was developed by the research group to assess the digital health literacy of community-dwelling older adults. Scale development applied a multistep approach as described by Devellis [42]. The first step is to clarify the concept connotation of digital health literacy through literature review. Based on the Transaction Model of eHealth literacy (TMeHL) proposed by Paige [21], the initial dimension of the scale was determined. With reference to the existing assessment tools [25, 31, 43–46], the item pool of digital health literacy assessment scale for community-dwelling older adults was formed. As the target population was the older adults in mainland China, all items were developed in Mandarin. Meanwhile, the inquiry was designed to be as brief and easy to understand as possible. The second step was to invite 22 experts with deep theoretical research and practical experience in the field of digital health and gerontological nursing to evaluate the contents of the scale by Delphi survey. After two rounds of consulting correspondence, the pre-test scale was formed. The third step was to conduct a pre-test among 30 community-dwelling older adults, all participants indicated that the wording and format of the items were understandable. So we did not adjust the expression and order of the scale after the pre-test. The fourth step was formal investigation. We recruited 457 community-dwelling older adults. Items were further screened by item analysis and exploratory factor analysis, and the reliability and the validity of the scale were verified by confirmatory factor analysis, criterion validity test, content validity test and internal consistency reliability test. Three common factors were extracted from the exploratory factor analysis, and the cumulative variance contribution rate was 78.726%. The results of confirmatory factor analysis showed that the model standardized regression coefficient was 0.645 ~ 0.910, and all the fitting indexes met the reference standard, indicating the model fitted well. Using the eHEALS as a criterion tool, the results showed that the total score and scores of each dimension of the scale were significantly positively correlated with those of the eHEALS, with the correlation coefficient of 0.455 ~ 0.948(*P* < 0.001), indicating the criterion validity was good. The item-level CVIs were 0.833~1.000, and the scale-level CVI was 0.967; the Cronbach's alpha was 0.941; the split-half reliability was 0.889; and the test–retest reliability after 2 weeks was 0.941. It usually took 10 to 20 min to complete the questionnaire in most older adults. The above results suggested that the scale had good reliability, validity and practicability. The formal scale included digital health information acquisition and evaluation ability (9 items), digital health information interaction ability (3 items) and digital health information application ability (3 items), with 3 dimensions and 15 items in total [47]. All items in the scale were closed questions and measured with 5-point Likert scale (1 = strongly disagree to 5 = strongly agree). The scale score was the sum of all items, with the total score ranging from 15 to 75. Higher scores indicate higher level of digital health literacy. The Cronbach's alpha in this study was 0.959.

### Data analysis

EpiData 3.1 was used for data entry and sorting, and SPSS 23.0 was used for analysis after double-checking by two researchers. Descriptive analysis of frequency, percentage, mean and standard deviation was conducted to determine the subjects' sociodemographic characteristics, Internet usage characteristics, attitude towards Internet health information and digital health literacy. T-test and ANOVA were used to compare the differences of digital health literacy of older adults with different sociodemographic characteristics, Internet usage characteristics and attitudes towards Internet health information. Multiple linear stepwise regression analysis was used to explore the influencing factors of digital health literacy of older adults. All statistical tests were two-tailed and statistical significance was set at 0.05.

## Results

### Sociodemographic characteristics of the respondents

The average age of participants was 70.93 years (SD 5.51), ranging from 65 to 88 years. More than half of participants had only junior high school education or below (388/572, 67.83%). About two-thirds of participants suffered from chronic diseases (420/572, 73.43%), and were quite/highly concerned about their health (424/572, 74.13%). Most of participants self-rated their health status as very good/good (236/572, 41.26%) or fair (231/572, 40.38%). For further details, see Table 1.

**Table 1:** The status of digital health literacy by different sociodemographic characteristics (*N* = 572)

Characteristic	n (%)	Digital health literacy		
		Mean (SD)	F or t	*P* value
Gender			0.594	0.553
Male	273 (47.73%)	37.58 (17.39)		
Female	299 (52.27%)	36.66 (19.75)		
Age (years)			19.637	< 0.001
65–69	283 (49.48%)	41.50 (18.46)		
70–74	144 (25.17%)	37.47 (18.46)		
75–79	92 (16.08%)	30.55 (16.28)		
≥ 80	53 (9.27%)	23.91 (14.55)		
Education level			72.835	< 0.001
Primary school or below	202 (35.31%)	24.64 (14.42)		
Junior high school	186 (32.52%)	39.82 (16.72)		
Senior high school	114 (19.93%)	46.65 (17.22)		
College and above	70 (12.24%)	50.24 (15.46)		
Living arrangement			8.924	< 0.001
Live with a spouse only	231 (40.38%)	40.61 (17.78)		
Live with children/grandchildren only	86 (15.03%)	29.64 (17.82)		
Live with spouse and children/grandchildren	191 (33.39%)	37.68 (19.12)		
Live alone or other	64 (11.19%)	32.69 (18.08)		
Marital status			3.621	< 0.001
Married	444 (77.62%)	38.60 (18.56)		
Unmarried/Divorced/Widowed	128 (22.38%)	31.89 (18.12)		
Monthly household income per capita (RMB)			42.579	< 0.001
< 1000	42 (7.34%)	22.02 (13.38)		
1000–2999	216 (37.76%)	30.37 (17.19)		
3000–4999	242 (42.31%)	41.97 (17.64)		
≥ 5000	72 (12.59%)	49.69 (15.16)		
Self-rated health status			22.379	< 0.001
Very poor/Poor	105 (18.36%)	27.04 (15.31)		
Fair	231 (40.38%)	37.56 (18.64)		
Good/Very good	236 (41.26%)	41.12 (18.44)		
Health concerns			7.921	< 0.001
Not concerned at all/Not quite concerned	41 (7.17%)	28.78 (18.42)		
General concerned	107 (18.71%)	33.57 (16.98)		
Quite concerned/Highly concerned	424 (74.13%)	38.79 (18.78)		
Chronic disease			0.803	0.422
No	152 (26.57%)	38.14 (19.66)		
Yes	420 (73.43%)	36.72 (18.29)		

### Internet usage characteristics and attitude towards Internet health information of the respondents

More than 2/3 of the participants used the Internet (389/572, 68.01%), and the average year of Internet usage was 6.74 years (SD 0.23), ranging from 1 to 26 years. More than half of participants surfed the Internet every day (361/572, 63.11%), but spent less time online, mostly within 1 h (243/572, 42.48%) and 1–2 h (171/572, 29.90%). Half of participants never/seldom asked for help actively from family members on acquiring Internet health information (296/572, 51.75%), and never/seldom received guidance passively from family members on acquiring Internet health information (277/572, 48.43%). In terms of attitude towards Internet health information, nearly half of participants thought that Internet health information was very useful/useful (237/572, 41.43%), but very difficult/difficult to use (287/572, 50.17%). They thought the Internet health information was very high-risk/high-risk (284/572, 49.65%), and not reliable at all/a little reliable (284/572, 49.65%). For further details, see Table 2.

**Table 2:** The status of digital health literacy by different internet-related characteristics (*N* = 572)

Characteristic	n (%)	Digital health literacy		
		Mean (SD)	F or t	*P* value
Duration of internet usage			450.972	< 0.001
0	183 (31.99%)	15.50 (2.22)		
1–5 year (s)	202 (35.31%)	41.54 (12.75)		
6–10 years	146 (25.52%)	52.42 (11.50)		
≥ 11 years	41 (7.17%)	57.02 (12.25)		
Frequency of internet usage			583.173	< 0.001
Never/Seldom	186 (32.52%)	15.45 (1.99)		
Several times a week	25 (4.37%)	34.76 (11.56)		
Every day	361 (63.11%)	48.41 (13.04)		
Time spent using the internet per day			286.262	< 0.001
< 1 h	243 (42.48%)	20.47 (11.10)		
1–2 h	171 (29.90%)	46.70 (11.88)		
3–4 h	101 (17.66%)	51.23 (12.53)		
≥ 5 h	57 (9.97%)	54.12 (13.06)		
frequency of asking for help actively			189.95	< 0.001
Never/Seldom	296 (51.75%)	25.71 (16.16)		
Sometimes	130 (22.73%)	49.07 (12.09)		
Often/Always	146 (25.52%)	49.52 (12.67)		
frequency of receiving guidance passively			220.901	< 0.001
Never/Seldom	277 (48.43%)	24.39 (15.43)		
Sometimes	140 (24.48%)	48.46 (12.53)		
Often/Always	155 (27.10%)	49.54 (12.57)		
Perceived usefulness			300.094	< 0.001
Not useful at all/Not useful	218 (38.11%)	20.12 (10.49)		
Unsure	117 (20.45%)	45.90 (12.82)		
Useful/Very useful	237 (41.43%)	48.37 (15.09)		
Perceived ease of use			408.686	< 0.001
Very difficult/Difficult	287 (50.17%)	23.00 (12.82)		
Unsure	48 (8.39%)	44.46 (11.64)		
Easy/Very easy	237 (41.43%)	52.68 (10.93)		
Perceived risk			247.122	< 0.001
Very low/Low	189 (33.04%)	51.39 (12.22)		
Unsure	99 (17.31%)	46.17 (14.33)		
High/Very high	284 (49.65%)	24.42 (14.33)		
Perceived reliability			273.213	< 0.001
Not at all/A little	284 (49.65%)	23.96 (14.00)		
Unsure	176 (30.77%)	49.81 (11.71)		
Somewhat/Mostly	112 (19.58%)	50.43 (14.05)		

### Digital health literacy among respondents

The mean score of digital health literacy of participants was 37.10 (SD 18.65), ranging from 15 to 74. The mean score of each item was 2.47 (SD 1.68), ranging from 1 to 5. This is in the negative end of the continuum, indicating that the community-dwelling older adults in this study have a relatively low perceived level of digital health literacy. The mean score of items in each dimension from high to low were as follows: digital health information acquisition and evaluation ability 2.89 (SD 1.71), digital health information interaction ability 2.18 (SD 1.56), digital health information application ability 1.51 (SD 1.03).

### Univariate analysis of digital health literacy of respondents

As shown in Table 1 and Table 2, respondents with different age, education level, residential status, marital status, monthly household income per capita, self-rated health status, degree of health concerns, duration of Internet usage, frequency of Internet usage, time spent using the Internet per day, frequency of asking for help actively and receiving guidance passively from family members on acquiring Internet health information, and perceived usefulness, perceived ease of use, perceived risk and perceived reliability towards Internet health information, the differences in digital health literacy were statistically significant (*P* < 0.01).

### Multiple linear regression analysis of digital health literacy of respondents

Taking digital health literacy score as dependent variable, 16 variables with statistical significance from univariate analysis as independent variables, the multiple linear regression analysis was conducted. The results showed that the regression model was significant (*F* = 222.510, *P* < 0.001), and 13 factors entered the regression equation, which could jointly account for 83.5% of the total variation, as shown in Table 3. Among them, education level, marital status, self-rated health status, degree of health concerns, duration of Internet usage, time spent using the Internet per day, frequency of Internet usage, frequency of receiving guidance passively from family members, perceived usefulness, perceived ease of use and perceived reliability were positively correlated with digital health literacy, while age and perceived risk were negatively correlated with digital health literacy.

**Table 3:** Multivariable linear regression predicting digital health literacy (*N* = 572)

Variable^a^	B (SE)	β	t	*P* value
Constant	4.62 (5.35)		0.86	0.388
Age	-0.20 (0.06)	-0.06	-3.20	0.001
Education level	1.95 (0.38)	0.11	5.14	< 0.001
Marital status	1.64 (0.80)	0.04	2.05	0.040
Self-rated health status	1.12 (0.45)	0.04	2.53	0.012
Health concerns	1.23 (0.54)	0.04	2.29	0.022
Duration of internet usage	0.59 (0.10)	0.15	5.90	< 0.001
Frequency of internet use	4.02 (0.67)	0.20	5.99	< 0.001
Time spent using the internet	2.63 (0.47)	0.14	5.63	< 0.001
Frequency of receiving guidance passively	2.08 (0.51)	0.09	4.11	< 0.001
Perceived usefulness	2.71 (0.55)	0.13	4.92	< 0.001
Perceived ease of use	3.32 (0.52)	0.17	6.35	< 0.001
Perceived risk	-1.07 (0.51)	-0.05	-2.11	0.035
Perceived reliability	2.78 (0.62)	0.12	4.48	< 0.001

^a^Model R^2^ = 0.838, Adjusted R^2^ = 0.835, *F* = 222.510, *P* < 0.001

## Discussion

As far as we know, our study is the first attempt to use the localized original assessment tool to evaluate the digital health literacy of community-dwelling older adults in China. Compared with the introduced universal scale used in previous studies, it is more in line with the current era context and cultural background, and the results are more targeted and accurate. Meanwhile, the influencing factors of digital health literacy of community-dwelling older adults was explored in depth by taking the objective factors (sociodemographic characteristics and previous Internet use experience) as well as the subjective factors (attitude towards Internet health information) into consideration. The research results not only enrich the existing body of knowledge in the field of digital health, but also provide valuable theoretical reference for the follow-up construction of gerontological digital health platform and the development of tailored digital health literacy intervention training programs for older adults in developing countries.

In this study, the overall digital health literacy of community-dwelling older adults was relatively low. Previous research results showed that there was a gap between the digital health literacy level of Chinese older adults and that of developed countries [14, 27]. One possible reason is that China gained full access to the Internet in 1994, later than many developed countries. Older adults failed to keep pace with the emergence and development of the Internet, some of whom were marginalized by the Internet. The number of Internet users in China aged 60 and over has tripled in the past five years, making them the fastest growing group [48, 49]. Age-related barriers to technology use have been gradually weakened by the high Internet penetration. At the same time, with the improvement of health self-management awareness, more and more older adults begin to seek health guidance from Internet and digital applications (e.g. WeChat, TikTok or Baidu) and then explore in practice. In this process, the ability to acquire and evaluate Internet health information has been gradually established [50]. However, the core of the digital media era is participation and interaction. Digital Health Literacy 2.0 emphasizes that users should not only be passive receivers, but also have the ability to actively participate in communication by interacting and sharing health-related information [21]. This study showed that most older adults had a low sense of media participation, and they were accustomed to acting as receivers rather than communicators, which was consistent with previous research results [19, 51]. The application ability of digital health literacy is the highest cognitive level of digital health literacy, which is cultivated by acquiring information from all lower-level digital health literacy dimensions (namely, acquisition and evaluation ability and interaction ability) [21]. In this study, the digital health application ability of the older adults was weak, most of whom lacked the ability to turn the Internet health knowledge into action and apply it to health self-management.

Further attempt was made to explore the multifaceted influencing factors of digital health literacy of community-dwelling older adults. The results showed that in terms of sociodemographic factors, age, education level, marital status, self-rated health status and degree of health concerns were important influencing factors. The younger, better educated and married older adults had higher digital health literacy, which was consistent with previous research results [14, 26]. The higher digital health literacy of the married older adults may be due to the fact that older adults with good family functions are more likely to get family emotional support, thus having more family involvement and guidance when using digital health services [52, 53]. The research by Paek et al. proved that communication with peers can enhance digital health literacy [54]. Older adults who perceive themselves to be good health and pay more attention to health have stronger self-care awareness and health information needs, and they are more likely to actively seek online health knowledge and skills and practice them in daily life [55]. These identified non-modifiable factors can be used to determine which individuals are at risk of poor digital health literacy, and to identify the vulnerable population that health professionals need to target. As the main healthcare provider, health professionals should carry out education and training programs for older adults to practice digital health resources. In order to ensure training effects, training programs must be tailored to the educational needs of older adults of different sociodemographic characteristics.

In terms of Internet usage factors, duration of Internet usage, frequency of Internet usage, time spent using the Internet per day, and the frequency of receiving guidance passively from family members were the influencing factors of the digital health literacy of older adults. As one of the six core literacy of digital health literacy, computer literacy is the foundation of digital health literacy cultivation [20]. Studies have shown that increasing the frequency of subjects using professional health websites is an effective intervention measure to improve digital health literacy [56, 57]. It was worth noting that frequency of asking for help actively from family members did not enter the regression equation, while the frequency of receiving guidance passively from family members entered the final regression equation. It was suggested that older adults were more inclined to learn passively than to seek guidance actively from their family members for obtaining Internet health information, and family members' teaching of acquiring Internet health information skills can improve the digital health knowledge and skills more effectively. This also provide a theoretical basis for the further intervention of digital health literacy for community-dwelling older adults at the family level. Under the background of Chinese social culture, older adults often live with their children and grandchildren. In this study, 48.42% of older adults lived with their spouses and children/grandchildren or live with children/grandchildren only, having the advantage of family education. It is necessary to actively carry out the digital back-feeding of the younger generations to the older generations on digital media knowledge, skills and associated pop culture and values [53], and encourage older adults to accumulate experience in practice to improve their digital health literacy.

In terms of the attitude towards Internet health information, perceived usefulness, ease of use, risk and reliability of Internet health information were influencing factors of digital health literacy of older adults. This result can be explained by the Technology Acceptance Model [58]. Older adults with high perceived usefulness, ease of use, reliability and low perceived risk of Internet health information may have more active attitude towards digital health services usage, which directly affects their behavior intention and leads to more positive usage behaviors, thus having higher digital health literacy. The study by Yang et al. also showed that the attitude towards Internet health information was the most important predictor of digital health literacy [50]. On the one hand, it is suggested that the benefits of digital health service and the skills to avoid risk should be further propagandized in the future. It is necessary to enhance older adults’ perception of the usefulness, ease of use and reliability of the digital health service, reduce the perception of risk, and help them accept the digital health service, so as to change the digital health behavior and improve the digital health literacy [59]. On the other hand, gerontological design of digital health services should be strengthened [60]. A series of measures should be taken to adapt to the physiological and psychological characteristics and media usage habits of older adults, such as enlarging fonts, simplifying operation interfaces and steps, and replacing technical terms with illustrated and plain languages. At the same time, the platform operation department should strengthen the quality control of Internet health information, improve the scientific nature of products and industry standardization, to create a safe and orderly Internet environment, so that older adults can better accept and use digital health services.

### Limitations and future research

There are still some limitations in this study. First of all, the digital health literacy assessment used the self-assessment scale, which inevitably led to self-reported bias and could not fully reflect the actual digital health literacy operation ability of the participants. In the future research, additional operational experiments should be considered for test. In addition, this study only investigated community-dwelling older adults in the main urban areas of Chongqing, China, as well as other limitations in the selection associated with the sample design already explained in the methodological section. Considering that the level of digital health literacy is closely related to the Internet penetration rate and the level of economic development [19], the research results can not represent the overall level of community-dwelling older adults in China. Therefore, additional large-scale studies in other regions are necessary to make the research results more representative.

## Conclusions

This study suggests that the digital health literacy level of community-dwelling older adults in Southwest China is relatively low, with the need to be improved urgently, among which the lack of interactive ability and application ability of digital health literacy deserved more attention from not only health professionals, but also public health policy makers and implementers. Meanwhile, a comprehensive intervention models linked by the government, community and family should be established. The government should strengthen policy guidance and the construction of the gerontological digital health platform. The community should regularly carry out standardized digital health education and training programs, and families should give full play to digital back-feeding advantages. The multi-party linkage will help improve the digital health literacy of older adults and enable them to fully enjoy the digital health dividend, and meanwhile spur the development of smart elderly care, thus better realizing the active aging.

## Supplementary Information

**Additional file 1.** Study questionnaire.

## Data Availability

The datasets used and/or analysed during the current study are not publicly available due to confidentiality as they include sensitive private information but are available from the corresponding author on reasonable request.

## Abbreviations

ANOVA: Analysis of variance
CVI: Content validity index
eHealth: Electronic health
eHEALS: EHealth literacy Scale
GDP: Gross domestic product
ICT: Information and communications technology
TMeHL: Transaction model of eHealth literacy
