# Supplementary material for: Current status and influencing factors of digital health literacy among community-dwelling older adults in Southwest China: a cross-sectional study
Source: BMC Public Health. 2022 May 17;22:996. doi: 10.1186/s12889-022-13378-4 (PMC9112275; doi:10.1186/s12889-022-13378-4)
Supplement: Supplementary file 1 — Additional file 1. Study questionnaire. [file 12889_2022_13378_MOESM1_ESM.doc]

**重庆市社区老年人数字健康素养调查问卷**

尊敬的老年朋友：

您好！随着互联网的普及与快速发展，网络已经成为大多数人获取医疗健康信息的重要渠道，数字化医疗平台的推广也给人们的生活提供了更多便利。我们课题组正在开展社区老年人数字健康素养评估及影响因素研究，该课题受国家社会科学基金（19XRK001）资助。我们想通过本问卷了解您使用电子设备上网查找健康信息应用于日常生活的情况，为进一步提升老年人数字健康素养水平，制定有针对性的干预策略提供科学依据。所有的资料仅用于研究用途，我们将严格保密。答案没有对错之分，您可以畅所欲言表达自己的观点，感谢您的热心参与和配合！

备注：问卷中提到的**电子设备**是指智能手机、数字电视、电脑、平板电脑、智能可穿戴设备等。

陆军军医大学护理学院 罗羽（教授）课题组

**一、个人基本信息**

以下是对您一般资料的调查，请根据您的具体情况在相应的选项上打“√”，如需进一步说明，请填写在“ ”上。

1.性别：£①男 £②女

2.年龄： 岁

3.文化程度：

£①未受教育 £②小学 £③初中 £④高中/技校/中专 £⑤大专及以上

4.居住状况：

£①与配偶同住 £②与（孙）子女同住 £③与配偶及（孙）子女同住 £④独居及其它

5.婚姻状况：£①未婚 £②已婚 £③离异 £④丧偶

6.家庭人均月收入：

£①<1000元 £②1000-2999元 £③3000-4999元 £④≥5000元

7.您自觉目前健康状况：£①非常差 £②差 £③一般 £④好 £⑤非常好

8.您对自己健康状况的关注程度：

£①不关注 £②不太关注 £③一般 £④比较关注 £⑤非常关注

9.您是否患有慢性疾病：£①无 £②有

**二、互联网使用情况和网络健康信息态度**

以下是对您互联网使用情况和网络健康信息态度的调查，请根据您的具体情况在相应的选项上打“√”，如需进一步说明，请填写在“ ”上。

1.您是否有上网的习惯：£①无 £②有，已使用互联网 年

2.您上网的频率为：

£①从不或极少 £②每周数次 £③几乎每天

3.您平均每天上网的时间为：

£①1小时以内 £②1-2小时 £③3-4小时 £④5-6小时 £⑤6小时以上

4.上网获取健康信息遇到困难时您是否向家人寻求帮助：

£①从不 £②很少 £③有时 £④经常 £⑤总是

5.您的家人是否曾经教您如何上网获取健康信息：

£①从不 £②很少 £③有时 £④经常 £⑤总是

6.您觉得网络上的健康信息是否有用：

£①毫无用处 £②不太有用 £③一般 £④比较有用 £⑤非常有用

7.您觉得通过网络获取健康信息是否容易：

£①非常困难 £②比较困难 £③一般 £④比较容易 £⑤非常容易

8.您觉得查找和应用网络健康信息所面临的风险：

£①非常小 £②比较小 £③一般 £④比较大 £⑤非常大

9.您对网络健康信息的信任程度是：

£①非常不相信 £②不太相信 £③一般 £④比较相信 £⑤非常相信

**三、社区老年人数字健康素养量表**

**以下是对您获取和使用网络健康信息方面的调查，请根据您的真实情况，在最符合的一项上打“√”：****（1=非常不符合，2=比较不符合，3=不确定，4=比较符合，5=非常符合）**

| **项 目** | **符合程度** |
| --- | --- |
| 1.我对学习网络上的健康知识或技能感兴趣 | £1 £2 £3 £4 £5 |
| 2.我会查看网络推送的健康信息 | £1 £2 £3 £4 £5 |
| 3.我会主动上网查找自己需要的健康信息 | £1 £2 £3 £4 £5 |
| 4.我能理解网络健康信息的内容 | £1 £2 £3 £4 £5 |
| 5.我能判断网络健康信息是否正确 | £1 £2 £3 £4 £5 |
| 6.我会通过咨询他人来判断网络健康信息的可靠性 | £1 £2 £3 £4 £5 |
| 7.我能从网络健康信息中筛选出自己需要的内容 | £1 £2 £3 £4 £5 |
| 8.即使是可信的、高质量的网络健康信息，我也会仔细考虑其对我的个人情况是否适用 | £1 £2 £3 £4 £5 |
| 9.在网络上查找和分享健康信息时我会注意保护自己和他人的隐私 | £1 £2 £3 £4 £5 |
| 10.我能在网络上分享或评论健康信息资源（如转发或评论朋友圈里的健康信息等） | £1 £2 £3 £4 £5 |
| 11.我能参与健康论坛或在线讨论（如微信群、QQ群的健康讨论等） | £1 £2 £3 £4 £5 |
| 12.我能参与健康话题的网络投票或调查 | £1 £2 £3 £4 £5 |
| 13.我能通过网络在线问诊，向医生提供诊断所需信息（如症状描述、既往病历、健康指标等） | £1 £2 £3 £4 £5 |
| 14.我会使用健康类网站或在线平台的相关功能（如预约挂号、在线缴费、查阅电子病历等） | £1 £2 £3 £4 £5 |
| 15.我会使用电子设备记录和管理个人健康指标（如血压、心率、体重等） | £1 £2 £3 £4 £5 |

*******************************************************************************

**调查到此结束，再次感谢您的支持和配合！**

**English language translation of the original survey** (the questionnaires in the English language version are the free translations of the authors, they have not been validated for research use)

**Questionnaire on Digital Health Literacy of the Community****-dwelling Older Adults in Chongqing**

Dear old friends,

With the popularity and rapid development of the Internet, the network has become an important channel for most people to obtain health information, and the promotion of digital health platform has also provided more convenience to people's lives. Our research group is carrying out a study on the digital health literacy assessment and influencing factors of the community-dwelling older adults, which is supported by the National Social Science Foundation (No.19XRK001) . Through this survey, we would like to know how you use electronic devices to access and application of health information in daily life, so as to provide a scientific basis for further improving digital health literacy of older adults and formulating targeted intervention strategies. All the information is for research purposes only and we will keep it strictly confidential. There is no right or wrong answer, you can express your views freely, thank you for your enthusiastic participation and cooperation!

Note: the electronic devices mentioned in the questionnaire refer to smartphone, digital television, desktop computer, laptop computer, tablet, smart wearable devices and so on.

Yours sincerely,

Pro. Yu Luo’s Research Group

School of Nursing, Army Medical University

**Part Ⅰ Basic information**

The following is a survey of your general information, please pick the appropriate option according to your specific situation, if you need further clarification, please fill in the blanks.

1.Gender: £①Male £②Female

2.Age:

3.Education Level:

£①Illiteracy £②Primary school £③Junior high school £④Senior high school £⑤College and above

4.Living condition:

£①Live with a spouse only £②Live with children/grandchildren only

£③Live with spouse and children/grandchildren £④Live alone or other

5.Marital status:

£①Unmarried £②Married £③Divorced £④Widowed

6.Monthly household income per capita (RMB):

£①＜1000 £②1000-2999 £③3000-4999 £④≥5000

7.In general, would you say your health is…?

£①Very poor £②Poor £③Fair £④Good £⑤Very good

8.Please indicate the degree of your concerns about your health：

£①Not concerned at all £②Not quite concerned £③General concerned

£④Quite concerned £⑤Highly concerned

9.Do you suffer from chronic diseases?

£①No £②Yes

**Part Ⅱ Internet usage and attitude towards Internet health information**

The following is a survey of your Internet use and attitude towards Internet health information. Please tick the appropriate box according to your specific situation. If you need further explanation, please fill in the blanks.

1.Do you use the Internet?

£①No £②Yes，I have been using the Internet for years.

2.How often do you use Internet?

£①Never or seldom £②Several times a week £③Almost every day

3.On average, how many hours do you use Internet per day?

£①Less than 1 hour £②1-2 hours £③3-4 hours £④5-6 hours £⑤More than 6 hours

4.How often do you turn to family members for help when you have trouble accessing health information online?

£①Never £②Seldom £③Sometimes £④Often £⑤Always

5.How often do your family members would impart the knowledge of getting online health information to you?

£①Never £②Seldom £③Sometimes £④Often £⑤Always

6.Please indicate the degree of your perceived usability of Internet health information:

£①Not useful at all £②Not useful £③Unsure £④Useful £⑤Very useful

7.Please indicate the degree of your perceived ease of use of Internet health information:

£①Very difficult £②Difficult £③Unsure £④ Easy £⑤Very easy

8.Please indicate the degree of your perceived risk of Internet health information:

£①Very low £②Low £③Unsure £④ High £⑤Very high

9.Please indicate the degree of your perceived reliability of online health information

£①Not at all £②A little £③Unsure £④ Somewhat £⑤Mostly

**Part Ⅲ Digital Health Literacy Assessment Scale for community-dwelling older adults**

I would like to ask you for your opinion and about your experience using the Internet for health information. For each statement, tell me which response best reflects your opinion and experience right now.（1=Strongly disagree，2=disagree，3=Undecided，4=Agree，5=Strongly agree）

| 1.I am interested in learning health knowledge or skills on the Internet. | £1 £2 £3 £4 £5 |
| --- | --- |
| 2.I will check the health information pushed on the Internet. | £1 £2 £3 £4 £5 |
| 3.I will search the Internet initiatively for the health information I need. | £1 £2 £3 £4 £5 |
| 4.I can understand the content of Internet health information. | £1 £2 £3 £4 £5 |
| 5.I can tell if Internet health information is correct. | £1 £2 £3 £4 £5 |
| 6.I judge the reliability of Internet health information by consulting others. | £1 £2 £3 £4 £5 |
| 7.I can filter out the content I need from the Internet health information. | £1 £2 £3 £4 £5 |
| 8.Even if it is credible, high-quality Internet health information, I will carefully consider whether it is appropriate for my personal situation. | £1 £2 £3 £4 £5 |
| 9.When I find and share health information on the Internet, I will pay attention to protecting the privacy of myself and others. | £1 £2 £3 £4 £5 |
| 10.I can share or comment on health information resources on the Internet (such as forwarding or commenting on health information in Wechat, etc.). | £1 £2 £3 £4 £5 |
| 11.I can participate in health forum or online discussion (such as the health discussion in Wechat group, QQ group, etc.) | £1 £2 £3 £4 £5 |
| 12.I can participate in online polls or surveys on health topics. | £1 £2 £3 £4 £5 |
| 13.I can consult online through the Internet to provide doctors with the information they need for diagnosis (such as symptom description, past medical records, health indicators, etc.) | £1 £2 £3 £4 £5 |
| 14.I can use the relevant functions of health websites or online health platforms (such as appointment registration, online payment, access to eHealth records, etc.) | £1 £2 £3 £4 £5 |
| 15.I can use electronic devices to record and manage personal health indicators (such as blood pressure, heart rate, weight, etc.) | £1 £2 £3 £4 £5 |

*******************************************************************************

**This is the end of survey. Thank you very much for your participation!**
